# Body mass index and absolute lymphocyte count predict disease-free survival in Korean breast cancer patients

**DOI:** 10.1038/s41416-021-01391-0

**Published:** 2021-04-19

**Authors:** Sung Min Ko, Janghee Lee, Soong June Bae, Su Jung Baik, Junghwan Ji, Dooreh Kim, Sung Gwe Ahn, Joon Jeong

**Affiliations:** 1 Department of Surgery, Gangnam Severance Hospital, Yonsei University College of Medicine, Seoul, Republic of Korea; 2 Department of Surgery, Sacred Heart Hospital, Hallym University, Dongtan, Republic of Korea; 3 Healthcare Research Team, Health Promotion Center, Gangnam Severance Hospital, Seoul, Republic of Korea

**Keywords:** Breast cancer, Risk factors

## Abstract

**Background:**

Our study evaluated the association between body mass index (BMI) and absolute lymphocyte count (ALC) in breast cancer patients and healthy females. Additionally, we determined the prognostic value of these factors in breast cancer.

**Methods:**

We retrospectively identified 1225 primary invasive breast cancer patients and 35,991 healthy females. Factors including BMI and complete blood count associated with disease-free survival (DFS) were assessed using a multi-variable Cox proportional hazard model.

**Results:**

BMI and ALC were positively correlated in breast cancer patients and healthy females (both *P* < 0.001). In multi-variable analysis, overweight or obese participants had worse DFS (hazards ratio [HR], 1.98; 95% confidence interval [CI], 1.34–2.92; *P* = 0.001) than underweight or normal-weight individuals, but patients with high ALC had better DFS than those with low ALC (HR, 0.43; 95% CI, 0.29–0.65; *P* < 0.001). After risk stratification according to BMI/ALC, high-risk patients with high BMI/low ALC had worse DFS than others (HR, 2.48; 95% CI, 1.70–3.62; *P* < 0.001).

**Conclusions:**

BMI and ALC were positive correlated, but their effect on breast cancer prognosis was opposite. Patients with high BMI/low ALC had worse DFS than others. Underlying mechanisms for effect of BMI/ALC on breast cancer prognosis should be studied in the future.

## Background

The global prevalence of breast cancer has substantially increased over recent decades.^1^ In South Korea, breast cancer is one of the most common malignancies among females, accounting for >2000 deaths in 2016.^2^ Since breast cancer is a heterogenic disease with several biological characteristics, various factors such as hormone receptors, human epidermal growth factor receptor 2 (HER2), grade and Ki-67 labelling index (LI) affect disease prognosis.^3,4^ Currently, significant efforts are being made to develop methods for the accurate prediction of breast cancer prognosis.

Obesity is another major global health concern; in South Korea, >60% women aged >40 years are overweight or obese.^5^ Obesity is an important risk factor for diabetes, cardiovascular disease and kidney disease; it has also been recently recognised as a risk factor for breast cancer.^6^ Although hormones, adipocytokines and inflammatory cytokines have been identified as potential mediators, the biological mechanisms that explain the association between obesity and breast cancer survival have not been conclusively established.^7^ Body mass index (BMI), calculated using body weight and height, is the most widely used measure for the degree of obesity.^8^

Inflammatory cells have an important role in breast cancer progression.^9^ Several parameters such as tumour-infiltrating lymphocytes (TILs) and neutrophil-to-lymphocyte ratio (NLR) can be used to assess immune response. Among them, peripheral blood cell count has been widely used as it is easy and cost-effective. Lymphocytes, including natural killer cells, T cells and B cells, are types of white blood cells (WBCs) that are found in the vertebrate immune system.^10^ T lymphocytes, involved in adaptive immunity, play a key role in tumour-specific immune response.^11^

Several researchers have suggested that an association between BMI and immune response are closely related.^12^ However, the clinical significance of this relationship has not been assessed in breast cancer patients. Therefore, our study aimed to identify the association between BMI and peripheral inflammatory cells in breast cancer patients and healthy females. Furthermore, we determined the effect of BMI and peripheral inflammatory cells on the prognosis of breast cancer patients.

## Methods

### Study population

We retrospectively identified 1225 primary invasive breast cancer patients from the Gangnam Severance Hospital breast cancer registry registered between January 2009 and December 2015. Patients’ clinicopathologic information was extracted from their medical records. All patients were South Korean; patients from western countries were excluded owing to different BMI criteria, and Asian patients with non-Korean parentage were not included in the registry. Patients underwent breast cancer surgery as curative treatment and received adjuvant therapy if needed. Patients who had received neoadjuvant chemotherapy were excluded as accurate evaluation of surgical pathology and disease stage was difficult in these patients. Patients with de novo stage IV cancer were also excluded.

Further, 35,991 healthy women were included from the Gangnam Severance Health Promotion Center registry between January 2007 and July 2020 to reconfirm the association between BMI and absolute lymphocyte count (ALC). Data on body weight, height and complete blood count (CBC) were collected. All women were South Korean and had never been diagnosed with or treated for malignant disease. Patients and healthy females excluded from the study were summarised in Supplementary Fig. S1.

Our study was approved by the institutional review board of Gangnam Severance Hospital (approval number: 3-2020-0207), which waived the requirement for written informed consent owing to the retrospective study design.

### Body mass index

Body weight and height of breast cancer patients were obtained on their first visit. If these were not measured at the first visit, measurements taken after admission for operation were used. All measurements were made prior to any treatment for breast cancer. The body weight and height of all healthy females were measured at their routine health examination. BMI was calculated as body weight in kilograms divided by the square of height in meters, defined by the World Health Organization (WHO).^8^ Participants were categorised using BMI cut-offs given in the WHO-Asia-Pacific classification.^13^ According to the Asian standards, people with BMI < 18.5 kg/m^2^ are considered underweight, and those with BMI ≥ 23.0 kg/m^2^ are considered overweight or obese.

### Complete blood count

Peripheral venous blood samples were collected from all breast cancer patients for preoperative evaluation prior to any treatment for breast tumour. For healthy females, same samples were obtained at their health check-up to evaluate haemoglobin levels and white blood cell counts. Venous blood was collected using 15 ml polypropylene tubes containing 10% ethylenediaminetetraacetic acid as an anticoagulant. All blood cell counts including WBC count, absolute neutrophil count (ANC), ALC, platelet count and monocyte count were assessed at the same institutional laboratory. NLR was calculated by dividing the absolute number of neutrophils by the absolute number of lymphocytes. Patients with WBC count ≥ 20.0 × 10^3^/µL were excluded from study due to the possibility of abnormal conditions including infectious disease at the time of examination. For risk stratification according to ALC, patients were divided into low and high groups based on the median value of ALC in the breast cancer cohort.

### Statistical analysis

The primary endpoint was disease-free survival (DFS), and additional analysis was performed for estimating overall survival (OS) as the secondary endpoint. DFS was defined as the time period between breast cancer surgery and tumour recurrence, secondary malignancy or any cause of death. Contralateral breast cancer was classified as a secondary malignancy and not metastasis. OS was defined as the time period from breast cancer surgery to death due to any reason. Statistical analyses were performed using SPSS version 25.0 (IBM Inc., Armonk, NY, USA). Difference between the groups was evaluated by the Chi-square test for categorical data and one-way ANOVA for continuous variables, after confirmation by Levene’s test for equality of variances. Kaplan–Meier survival estimates were used to compare DFS, and the group differences in the survival curves were analysed using the log-rank test. Univariable and multi-variable Cox proportional hazard models were used to identify variables associated with DFS and OS and perform subgroup analysis with HR and corresponding 95% CI. All statistical tests were two sided, and a *P*-value <0.050 was considered statistically significant.

## Results

### Association between BMI and ALC

**Fig. 1:**
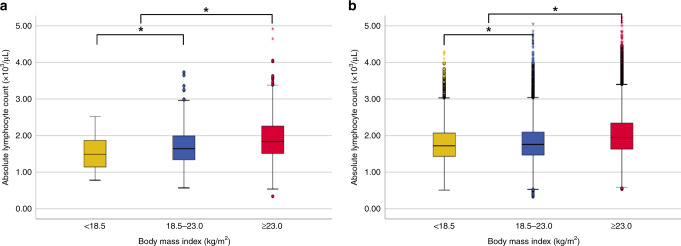
Boxplot comparing ALC according to BMI: **a** ALC according to BMI in breast cancer patients, **b** ALC according to BMI in healthy females. ALC absolute lymphocyte count, BMI body mass index. *P < 0.050.

In the breast cancer cohort, ALC was positive correlated with BMI. The average ALC was 1.81 × 10^3^/µL; ALC was the lowest in underweight patients and the highest in overweight or obese patients (*P* < 0.001; Fig. 1a). WBC, platelet count and monocyte count was increased in patients with overweight or obese compared to patients with normal weight. (WBC, *P* < 0.001; platelet, *P* < 0.001; monocyte, *P* = 0.001; Supplementary Fig. S2). However, ANC was not correlated with BMI (*P* = 0.073). NLR was significantly lower in overweight or obese patients than in underweight patients (*P* = 0.025), but there was no significant difference compared with the NLR of normal-weight patients (*P* = 0.641).

Next, we analysed CBC according to BMI in healthy females to compare results with those of breast cancer patients. The average age of healthy females was 47.0 years, and >50% had a normal body weight. 38.2% women were overweight or obese. In the healthy female cohort, BMI and ALC were also positively correlated. ALC was significantly higher in overweight or obese women than in underweight and normal-weight women (*P* < 0.001; Fig. 1b). The average ALC for healthy females was 1.89 × 10^3^/µL. Other blood cell counts such as WBC, platelet count, monocyte count and ANC also had a positive correlation with BMI (all *P* < 0.001; Supplementary Fig. S3). NLR was lower in overweight or obese women than in normal-weight or underweight women (*P* < 0.001). In addition, healthy females had significantly higher ALC than breast cancer patients (Supplementary Table S1). In contrast, WBC and ANC were higher in breast cancer patients than healthy females. There was no statistical difference in platelet and monocyte between the two groups.

### Baseline characteristics of breast cancer patients

Table 1 summarises the clinicopathologic characteristics of patients according to BMI. There were 51.4% overweight or obese patients and 4% underweight patients. The average patient age was 51.4 years; the average age of patients tended to increase with the increasing BMI. Additionally, a high proportion of patients with high BMI had tumours measuring >20 mm. Other features including grade, oestrogen receptor (ER)/progesterone receptor (PR)/HER2 status, Ki-67 LI, and the number of positive axillary lymph nodes did not differ with BMI. Type of breast and axillary surgery and adjuvant therapy also did not differ with BMI (Supplementary Table S2).

**Table 1:** Clinicopathologic features of breast cancer patients.

	Body mass index, kg/m^2^ (%)				*P*-value
	All patients	<18.5	18.5–23.0	≥23.0	
Total	1,225 (100)	49 (4.0)	546 (44.6)	630 (51.4)	
Age, average (range), years	51.4 (24–87)	43.9 (26–67)	48.5 (24–87)	54.6 (28–87)	<0.001
Histologic grade					0.157
Low	250 (20.7)	11 (22.4)	125 (22.9)	114 (18.1)	
Intermediate	609 (50.4)	20 (40.8)	260 (47.6)	329 (52.2)	
High	350 (28.5)	18 (36.7)	155 (28.4)	177 (28.1)	
Unknown	16 (1.3)	0 (0.0)	6 (1.1)	10 (1.6)	
Nuclear grade					0.263
Low	122 (10.0)	8 (16.3)	61 (11.2)	53 (8.4)	
Intermediate	621 (50.7)	24 (49.0)	267 (48.9)	330 (52.4)	
High	467 (38.1)	17 (34.7)	212 (38.8)	238 (37.8)	
Unknown	15 (1.2)	0 (0.0)	6 (1.1)	9 (1.4)	
Oestrogen receptor					0.778
Negative	355 (29.0)	12 (24.5)	159 (29.1)	184 (29.2)	
Positive	870 (71.0)	37 (75.5)	387 (70.7)	446 (70.8)	
Progesterone receptor					0.726
Negative	473 (38.6)	18 (36.7)	205 (37.5)	250 (39.7)	
Positive	752 (61.3)	31 (63.3)	341 (62.5)	380 (60.3)	
HER2					0.921
Negative	873 (71.2)	34 (69.4)	391 (71.5)	448 (71.1)	
Positive	306 (25.0)	12 (24.5)	133 (24.5)	161 (25.6)	
Unknown	46 (3.8)	3 (6.1)	22 (4.0)	21 (3.3)	
Ki-67 LI, %					0.370
<14	671 (54.8)	27 (54.0)	287 (52.5)	357 (56.7)	
≥14	554 (45.2)	22 (44.9)	259 (47.4)	273 (43.3)	
Tumour size, mm					0.001
≤20	801 (65.4)	36 (73.5)	385 (70.5)	380 (60.3)	
>20	424 (34.6)	13 (26.5)	161 (29.5)	250 (39.7)	
Positive lymph node, count					0.216
0	842 (68.7)	40 (81.6)	373 (68.3)	429 (68.1)	
1–3	296 (24.2)	6 (12.2)	139 (25.5)	151 (24.0)	
≥4	87 (7.1)	3 (6.1)	34 (6.2)	50 (7.9)	

*HER2* human epidermal growth factor receptor 2, *LI* labelling index.

### BMI and ALC as prognostic factors for DFS and OS

The 5-year DFS for all patients was 91.9%. The 5-year distant recurrence and locoregional recurrence rates were 3.7% and 2.0%, respectively. The median follow-up period was 70 months. There were 152 events in 126 patients during follow-up. There were 87 recurrence, 51 secondary primary malignancy and 14 death events. Two events of recurrence or secondary malignancy occurred together in 14 patients, and two patients died without any recurrence and secondary tumour. Six patients developed contralateral breast cancer. Details of disease events have been summarised in Supplementary Table S3. For the analysis, BMI was divided into three groups—underweight (<18.5 kg/m^2^), normal weight (18.5–23.0 kg/m^2^) and overweight or obese (≥23.0 kg/m^2^), and continuous variables were used for blood cell counts. In univariable analysis, overweight or obesity, but not underweight, was a risk factor for DFS (hazards ratio [HR], 1.57; 95% confidence interval [CI], 1.09–2.26; *P* = 0.016; Table 2). Among blood cell counts, patients with high ALC had significantly better DFS than those with low ALC (HR, 0.52; 95% CI, 0.37–0.74; *P* < 0.001). NLR was also statistically significant (*P* = 0.001), but the associated HR was 1.08, suggesting minimal impact on prognosis. Other known prognostic factors including histologic grade, ER/PR/HER2 status, Ki-67 LI, tumour size and the number of positive lymph nodes (≥4) were also significant in univariable analysis. In multi-variable analysis, high BMI was associated with worse DFS (HR, 1.98; 95% CI, 1.34–2.92; *P* = 0.001) and high ALC was a good prognostic factor (HR, 0.43; 95% CI, 0.29–0.65; *P* < 0.001). However, excluding large tumour size (>20 mm; HR, 1.47; 95% CI, 1.00–2.18; *P* = 0.050) and high Ki-67 LI (HR, 1.94; 95% CI, 1.22–3.07; *P* = 0.005), other factors were not significant in multi-variable analysis. The Kaplan–Meier survival curves also revealed that the high BMI group had worse DFS than the normal and underweight groups (log-rank *P* = 0.025; Supplementary Fig. S4A). Based on the median ALC of 1.74 × 10^3^/uL in the breast cancer cohort, we divided patients into the low ALC group (*N* = 615) and the high ALC group (*N* = 610). Patients with high ALC had better DFS than those with low ALC according to the Kaplan–Meier survival analysis (log-rank *P* = 0.018; Supplementary Fig. S4B). However, BMI and ALC were not risk factors for OS (Supplementary Table S4). Ki-67 LI and tumour size were the only prognostic factors associated with OS in multi-variable analysis.

**Table 2:** Univariable and multi-variable Cox regression analysis for DFS.

	Univariable analysis		Multi-variable analysis	
	HR (95% CI)	*P-*value	HR (95% CI)	*P*-value
Age, years^a^	1.00 (0.99–1.02)	0.775		
BMI, kg/m^2^				
18.5–23.0	Ref. ^b^		Ref.	
<18.5	0.76 (0.24–2.45)	0.644	0.50 (0.12–2.06)	0.334
≥23.0	1.57 (1.09–2.26)	0.016	1.98 (1.34–2.92)	0.001
WBC count, ×10^3^/uL^a^	0.99 (0.89–1.09)	0.764		
ALC, ×10^3^/uL^a^	0.52 (0.37–0.74)	<0.001	0.43 (0.29–0.65)	<0.001
ANC, ×10^3^/uL^a^	1.06 (0.96–1.17)	0.270		
NLR, ×10^3^/uL^a^	1.09 (1.04–1.14)	0.001	1.01 (0.94–1.07)	0.881
Platelet count, ×10^3^/uL^a^	1.00 (0.99–1.00)	0.785		
Monocyte count, ×10^3^/uL^a^	1.72 (0.38–7.80)	0.484		
Histologic grade				
Low	Ref.		Ref.	
Intermediate	1.51 (0.89–2.55)	0.128	0.94 (0.54–1.65)	0.831
High	2.03 (1.18–3.50)	0.011	0.85 (0.44–1.65)	0.625
Nuclear grade				
Low	Ref.			
Intermediate	1.29 (0.64–2.60)	0.486		
High	1.94 (0.96–3.91)	0.065		
Oestrogen receptor				
Negative	Ref.		Ref.	
Positive	0.54 (0.38–0.78)	0.001	0.82 (0.46–1.49)	0.516
Progesterone receptor				
Negative	Ref.		Ref.	
Positive	0.54 (0.38–0.76)	<0.001	0.89 (0.50–1.56)	0.678
HER2				
Negative	Ref.		Ref.	
Positive	1.59 (1.10–2.31)	0.015	1.19 (0.80–1.79)	0.388
Ki-67 LI, %				
<14	Ref.		Ref.	
≥14	2.40 (1.67–3.45)	<0.001	1.94 (1.22–3.07)	0.005
Tumour size, mm				
≤20	Ref.		Ref.	
>20	2.12 (1.50–3.01)	<0.001	1.47 (1.00–2.18)	0.050
Positive lymph node, count				
0	Ref.		Ref.	
1–3	1.16 (0.77–1.76)	0.471	1.00 (0.65–1.55)	0.992
≥4	2.17 (1.27–3.73)	0.005	1.30 (0.72–2.34)	0.385

*ALC* absolute lymphocyte count, *ANC* absolute neutrophil count, *BMI* body mass index, *CI* confidence interval, *DFS* disease-free survival, *HER2* human epidermal growth factor receptor 2, *HR* hazard ratio, *LI* labelling index, *NLR* neutrophil-to-lymphocyte ratio, *WBC* white blood cell.

^a^Continuous variable.

^b^Reference value.

### Risk stratification according to BMI/ALC

**Fig. 2:**
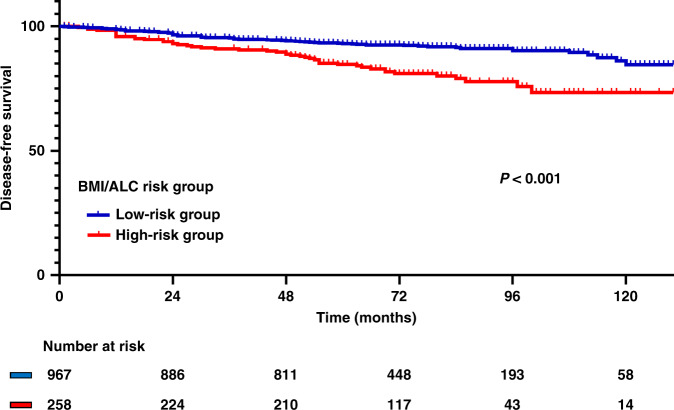
Kaplan–Meier survival curve for DFS according to the BMI/ALC risk stratification groups (log-rank *P* <  0.001). ALC absolute lymphocyte count, BMI body mass index, DFS disease-free survival.

We scored patients according to BMI/ALC and divided them into two risk stratification groups (low and high). If patients were overweight or obese and had low ALC, they were classified into the high-risk group (*N* = 258). Conversely, if patients were normal or underweight or had high ALC, they were classified into the low-risk group (*N* = 967). The Kaplan–Meier survival curves showed that the high-risk group had poorer prognosis than the low-risk group (log-rank *P* < 0.001; Fig. 2). The high-risk group also had worse DFS than the low-risk group according to the multi-variable Cox regression hazard model (HR, 2.48; 95% CI, 1.70–3.62; *P* < 0.001; Table 3). However, OS did not differ between the risk stratification groups (log-rank *P* = 0.528; Supplementary Fig. S5).

**Table 3:** Multi-variable Cox regression analysis for DFS using the BMI/ALC risk stratification group.

	HR (95% CI)	*P*-value
BMI/ALC risk group		
Low	Ref. ^b^	
High	2.48 (1.70–3.62)	<0.001
NLR, ×10^3^/uL^a^	1.04 (0.98–1.10)	0.161
Histologic grade		
Low	Ref.	
Intermediate	0.98 (0.56–1.71)	0.940
High	0.86 (0.44–1.67)	0.654
Oestrogen receptor		
Negative	Ref.	
Positive	0.79 (0.43–1.43)	0.428
Progesterone receptor		
Negative	Ref.	
Positive	0.95 (0.53–1.68)	0.855
HER2		
Negative	Ref.	
Positive	1.17 (0.78–1.76)	0.450
Ki-67 LI, %		
<14	Ref.	
≥14	1.94 (1.22–3.07)	0.005
Tumour size, mm		
≤20	Ref.	
>20	1.46 (0.98–2.15)	0.060
Positive lymph node, count		
0	Ref.	
1–3	0.98 (0.63–1.53)	0.943
≥4	1.35 (0.75–2.43)	0.321

*ALC* absolute lymphocyte count, *BMI* body mass index, *CI* confidence interval, *DFS* disease-free survival, *HER2* human epidermal growth factor receptor 2, *HR* hazard ratio, *NLR* neutrophil-to-lymphocyte ratio, *LI* labelling index.

^a^Continuous variable.

^b^Reference value.

**Fig. 3:**
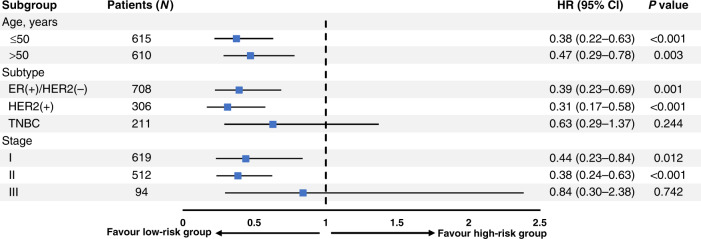
Subgroup analysis of DFS according to the BMI/ALC risk stratification groups. ALC absolute lymphocyte count, BMI body mass index, CI confidence interval, DFS disease-free survival, ER oestrogen receptor, HER2 human epidermal growth factor receptor 2, HR hazard ratio, TNBC triple-negative breast cancer.

In the subgroup analysis, BMI/ALC was a significant risk factor in both young (≤50 yrs) and old (>50 yrs) breast cancer patients (young: HR, 0.38; 95% CI, 0.22–0.63; *P* < 0.001; old: HR, 0.47; 95% CI, 0.29–0.78; *P* = 0.003; Fig. 3). In addition, the low-risk group had significantly better DFS for HER2-positive tumour than the high-risk group (HR, 0.31; 95% CI, 0.17–0.58; *P* < 0.001). There was also a difference in DFS for ER-positive/HER2-negative breast cancer between groups (HR, 0.39; 95% CI, 0.23–0.69; *P* = 0.001). However, DFS for triple-negative breast cancer did not significantly differ between groups (*P* = 0.244), although the HRs were different. Furthermore, the BMI/ALC risk groups were better validated for early breast cancer than for advanced breast cancer (stage I: HR, 0.44; 95% CI, 0.23–0.84; *P* = 0.012; stage II: HR, 0.38; 95% CI, 0.24–0.63; *P* < 0.001).

## Discussion

Our study found that BMI and ALC were positive correlated not only in breast cancer patients but also in healthy females, but their effect on breast cancer prognosis was opposite. Being overweight or obese adversely affected DFS, but patients with high ALC had better DFS than those with low ALC. After BMI/ALC risk stratification, high-risk patients with high BMI/low ALC had significantly worse DFS than low-risk patients.

Recent reports have suggested that obesity is a risk factor for various metabolic diseases; it is also associated with the risk of breast cancer. The Predicting Risk of Cancer at Screening (PROCAS) study in United Kingdom found that weight gain in adults is a risk factor for breast cancer, especially for those with BMI < 23.4 kg/m^2^ at 20 years of age.^14^ Of the 47,042 non-breast cancer patients followed up for a median of 5.6 years, 1142 were diagnosed with breast cancer. Therefore, weight management is important for preventing breast cancer and metabolic diseases, such as diabetes and cardiovascular disease.

Body weight is also an important prognostic factor in breast cancer patients. Chan et al. argued that obesity is associated with poor OS and breast cancer-specific survival (BCSS) in breast cancer patients in their systemic literature review of 82 follow-up studies.^15^ In this meta-analysis, the relative risk (RR) of total mortality in obese women was 1.75 and 1.34 for premenopausal and postmenopausal breast cancer, respectively. Our study findings were also similar to those of previous studies. In our results, DFS of breast cancer patients is worse if BMI is high.

Unlike overweight or obese patients, prognosis of underweight patients did not differ from normal-weight patients. Previous studies in South Korea have reported that underweight women may have risk factors for breast cancer such as early menarche and nulliparity, leading to worse OS and BCSS.^16,17^ However, in our study, underweight was not a risk factor for DFS and OS. Additional investigations, including more numbers of underweight patients than our study, are needed since the proportion of underweight patients in our cohort was very small.

The importance of immune responses in breast cancer is gradually being recognised. Several studies have found that breast tumours with high TILs have better prognosis than those with low TILs. In addition, peripheral lymphocytes and neutrophils can migrate toward a tumour site and infiltrate the tumour microenvironment.^18–21^ Thus, research on immunologic markers in peripheral blood, such as NLR, platelet-to-lymphocyte ratio (PLR) and lymphocyte-to-monocyte ratio is being actively conducted.^22,23^ However, there is a lack of data on ALC as a prognostic marker in breast cancer. Although the reported findings were discordant, previous studies have argued that ALC can predict DFS and mortality.^24,25^ In addition, some researchers have suggested that ALC is superior to NLR and PLR for predicting progression-free survival in breast cancer.^26^ In our study, ALC was a good predictor of DFS in breast cancer patients. Furthermore, we demonstrated that ALC is more strongly associated with prognosis than other blood parameters including NLR.

We divided the patients into two groups using the median ALC (1.74 × 10^3^/uL), which was consistent with the ALC reported in previous studies (1.5–1.8 × 10^3^/uL).^24–26^ However, lymphopenia is defined as ALC < 1.0 × 10^3^/uL, a much lower level; hence, low ALC in breast cancer patients and lymphopenia have different implications. Therefore, further research on new cut-off values that can be used to demarcate low and high ALC for oncology studies is needed. In addition, lymphocytes occasionally have opposing functions. CD8+ cytotoxic T lymphocytes increase anti-tumour immunity, and CD4+ helper T cells play critical roles in adaptive immune response along with B lymphocytes and CD8+ cytotoxic T cells.^27–29^ However, exhausted CD8+ T lymphocytes and regulatory T cells (T_reg_; subset of CD4+ T lymphocyte) suppress anti-tumour immunity.^30,31^ Therefore, the immune response to breast tumour could vary depending on the composition of lymphocytes, which ultimately affects prognosis. Further investigation of the association between peripheral lymphocyte composition and survival is needed.

To our knowledge, this is the first study to simultaneously analyse BMI and ALC in breast cancer patients. Previous experimental studies have suggested that obesity results in hypertrophy of adipose tissue. The release of adipocytokines leads to excessive immune cell recruitment with lymphocyte predominance.^12,32^ Hypertrophic adipocytes increase the expression of pro-inflammatory cytokines and activate CD8+ cytotoxic T lymphocytes and CD4+ helper T cells, but not T_reg_ lymphocytes.^33^ Our study demonstrated that these experimental results were consistent with clinical observations in breast cancer patients. In addition, the positive correlation between BMI and ALC was reconfirmed in a large healthy population. Therefore, we concluded that BMI and ALC are closely associated. Particularly, the results regarding obesity and lymphocyte for healthy women may be relevant to other fields of research. They may also be used to determine the effect of BMI/ALC on breast cancer development in further studies. Validation of the correlation between breast cancer occurrence and BMI/ALC in normal population might provide more specific information on the risk of breast cancer.

Our results suggest that increased BMI and ALC were not only independent prognostic factors, but their effect on breast cancer prognosis was opposite. ALC increased with the increasing BMI, but patients with high BMI/low ALC had worse DFS than those who did not. However, additional studies are necessary as the association between obesity and immune response and mechanisms underlying the effect of BMI/ALC on breast cancer prognosis are not fully understood.

Our study has some limitations. There was scope for selection bias owing to its retrospective design. Since patients with advanced breast cancer were usually treated with neoadjuvant chemotherapy in our institution, most participants in our cohort were early breast cancer patients. However, our finding that BMI and ALC are risk factors for DFS even in early breast cancer with a comparatively low recurrence rate may be meaningful. It was difficult to accurately analyse the effect of underweight on breast cancer prognosis owing to the small proportion of underweight patients in our cohort. Peripheral inflammatory cells can be affected by past history of patients including chronic disease, alcohol consumption and smoking habit. Our study did not take these variables into account. Analysing them together in a future study could provide a clearer evidence for the prevention of breast cancer. Furthermore, measuring obesity using various techniques such as body fat measurement in addition to BMI may helpful in evaluating the association between breast cancer and obesity. Because body weight and CBC data were recorded only at the time of diagnosis, serial changes in these parameters could not be analysed. Thus, we cannot ascertain if weight management after diagnosis is related to breast cancer prognosis.

In conclusion, patients with high BMI/low ALC had worse DFS than other groups. Therefore, these high-risk patients may require more careful observation and aggressive treatment. Additional studies are needed to delineate the underlying mechanisms by which BMI/ALC affect breast cancer prognosis.

## Supplementary information

Supplementary files

## Data Availability

The datasets used and/or analysed during the current study are available from the corresponding author on reasonable request.
