# Supplementary material for: Body mass index and absolute lymphocyte count predict disease-free survival in Korean breast cancer patients
Source: Br J Cancer. 2021 Apr 19;125(1):119–25. doi: 10.1038/s41416-021-01391-0 (PMC8257741; doi:10.1038/s41416-021-01391-0)
Supplement: Supplementary file 1 — Supplementary files [file 41416_2021_1391_MOESM1_ESM.docx]

**
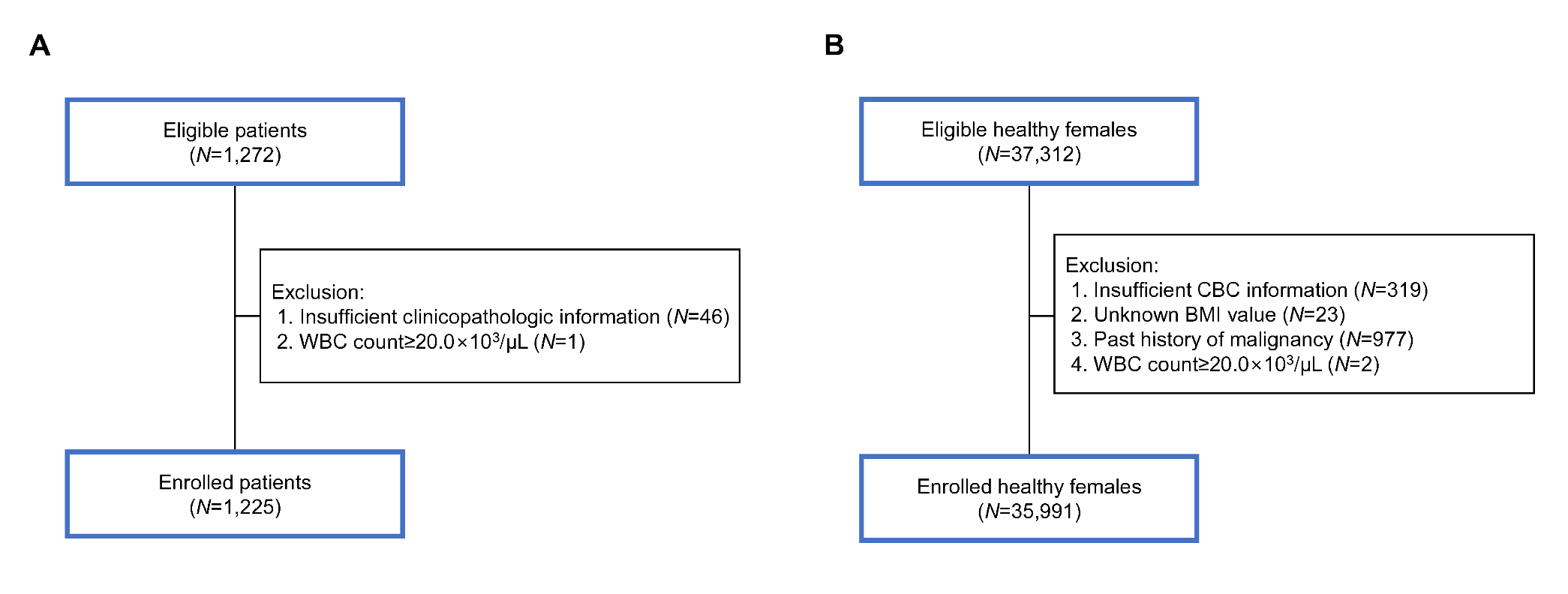
**

**Supplementary Figure S1.** Flowchart of enrolled breast cancer patients and healthy females: (A) Breast cancer patients, (B) Healthy females

WBC, white blood cell; CBC, complete blood count; BMI, body mass index

**
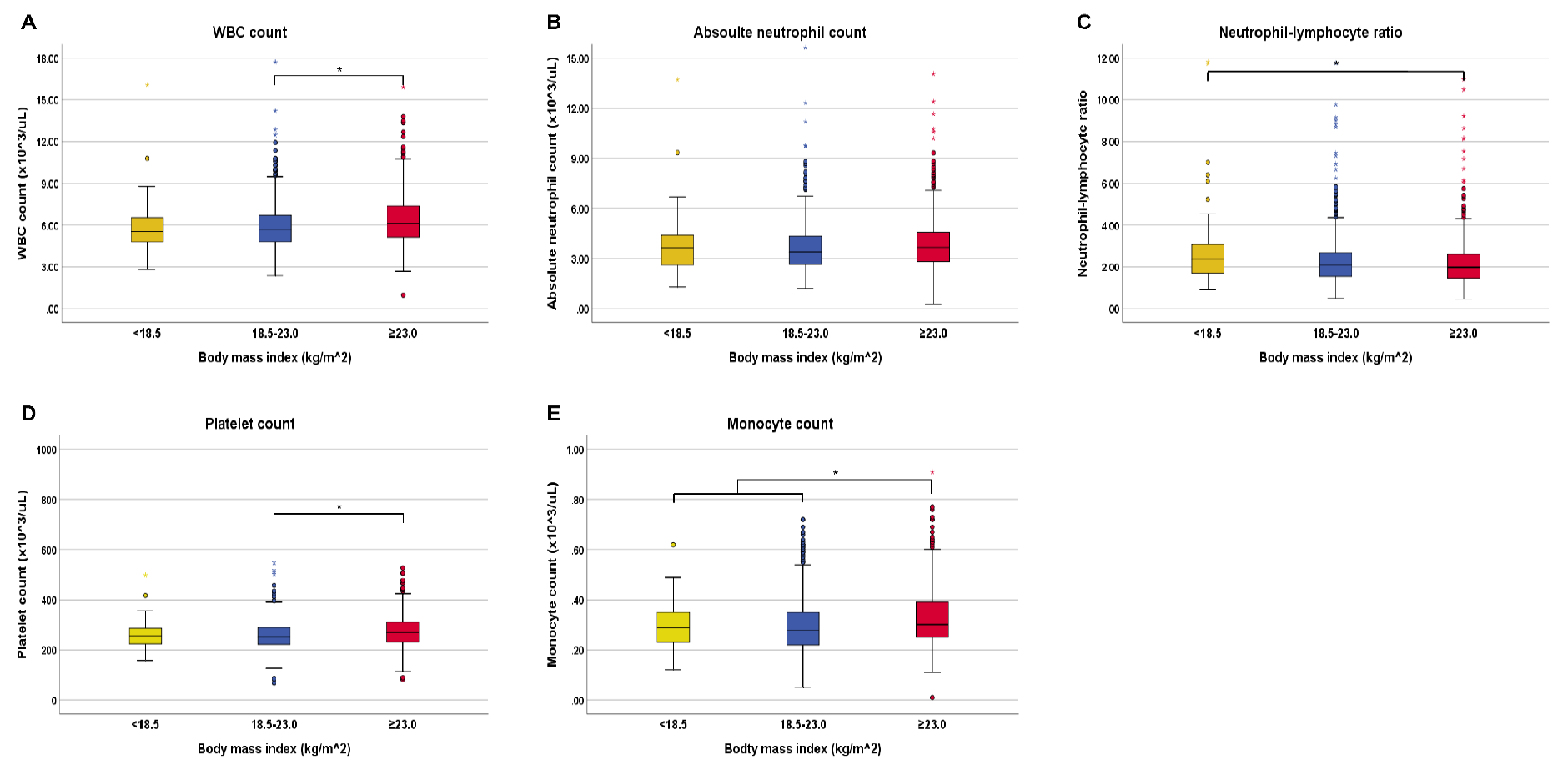
**

**Supplementary Figure S2.** Box plot comparing CBC results according to BMI in breast cancer patients: (A) WBC count, (B) ANC, (C) NLR, (D) Platelet count, (E) Monocyte count

ANC, absolute neutrophil count; BMI, body mass index; CBC, complete blood count; NLR, neutrophil-to-lymphocyte ratio; WBC, white blood cell; **P*<0.050

**
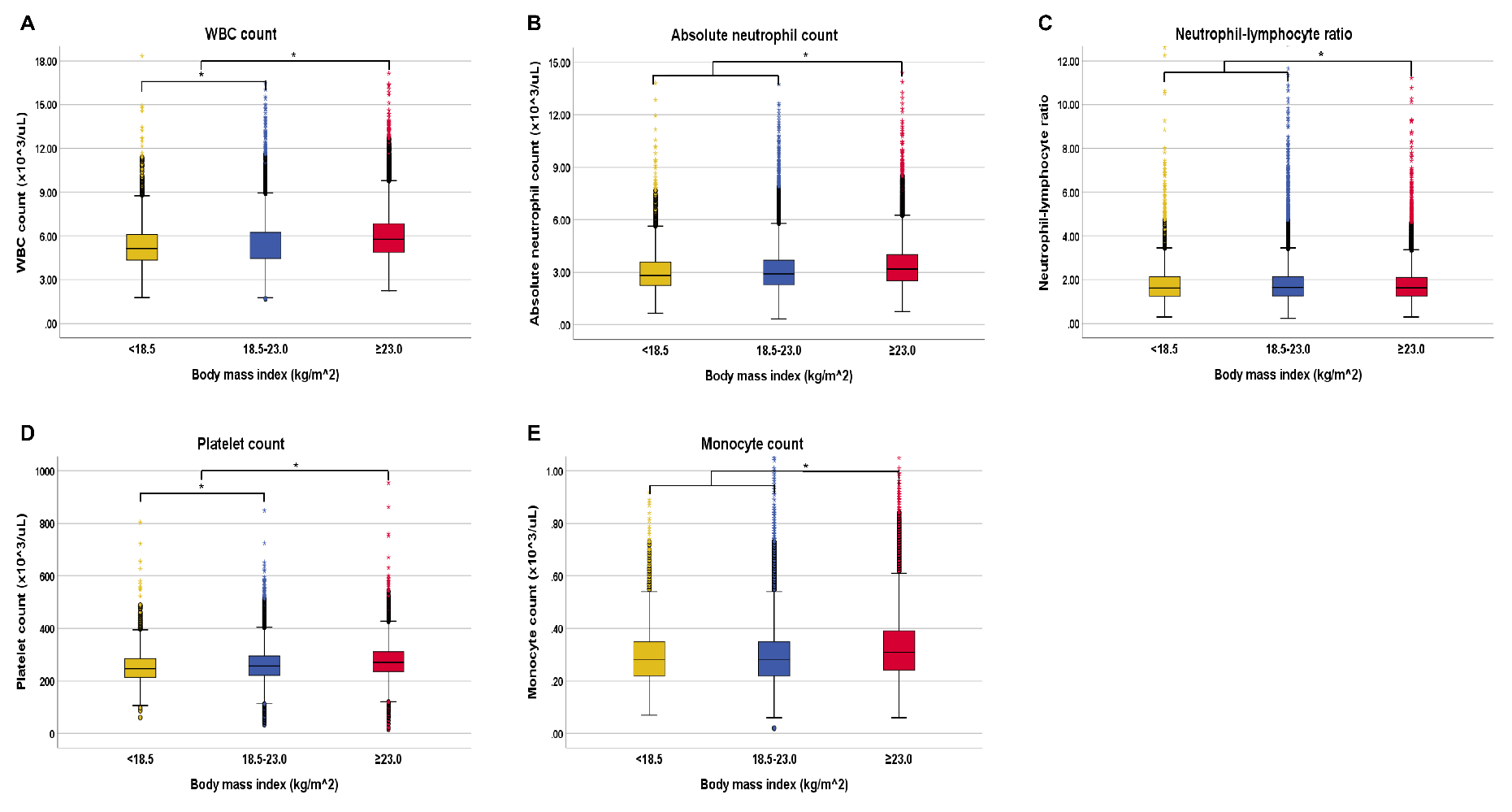
**

**Supplementary Figure S3.** Box plot comparing CBC results according to BMI in healthy females: (A) WBC count, (B) ANC, (C) NLR, (D) Platelet count, (E) Monocyte count

ANC, absolute neutrophil count; BMI, body mass index; CBC, complete blood count; NLR, neutrophil-to-lymphocyte ratio; WBC, white blood cell; **P*<0.050


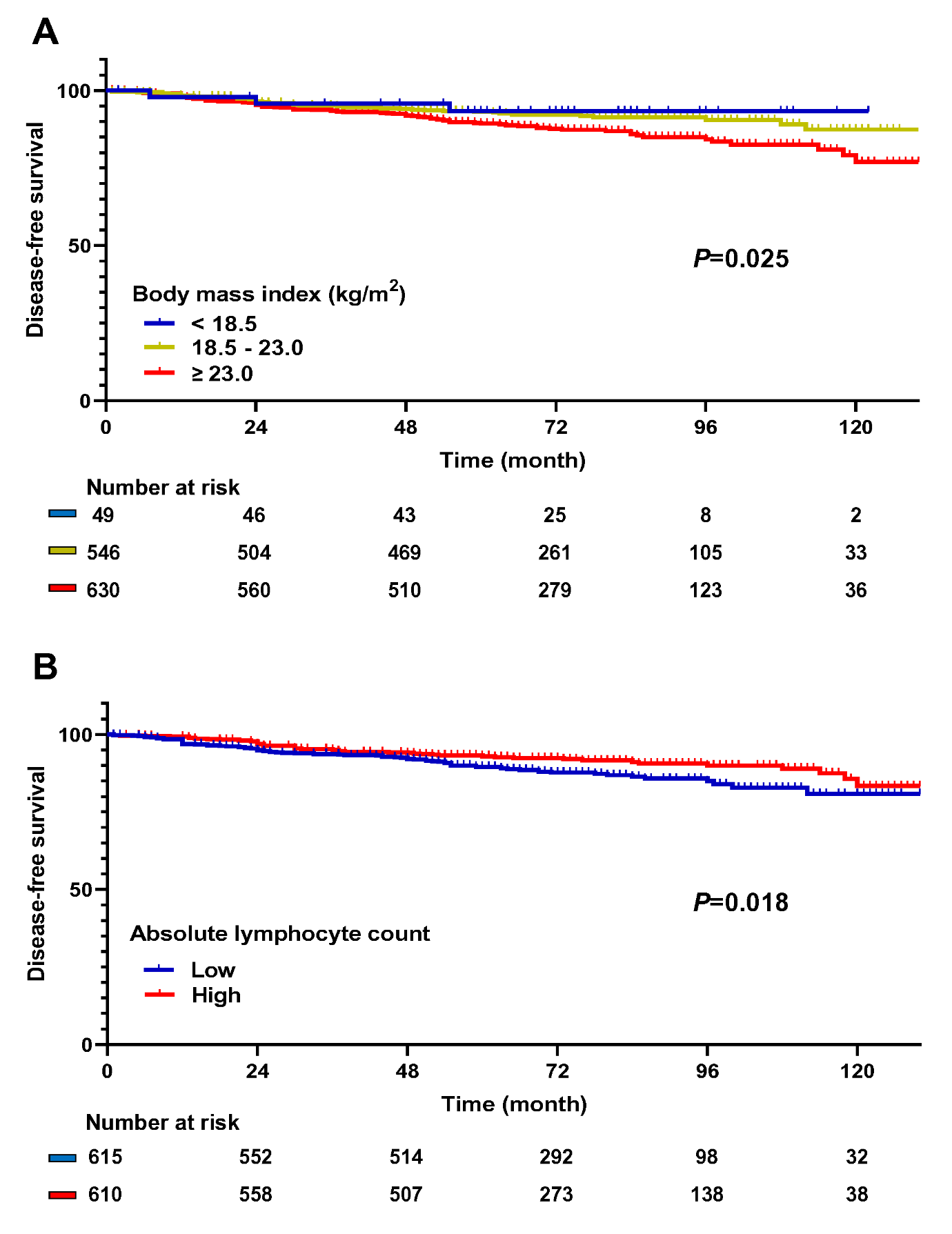


**Supplementary Figure S4.** Kaplan–Meier survival curve for DFS according to BMI and ALC: (A) BMI (log-rank *P*=0.025), (B) ALC (log-rank *P*=0.018)

ALC, absolute lymphocyte count; BMI, body mass index; DFS, disease-free survival


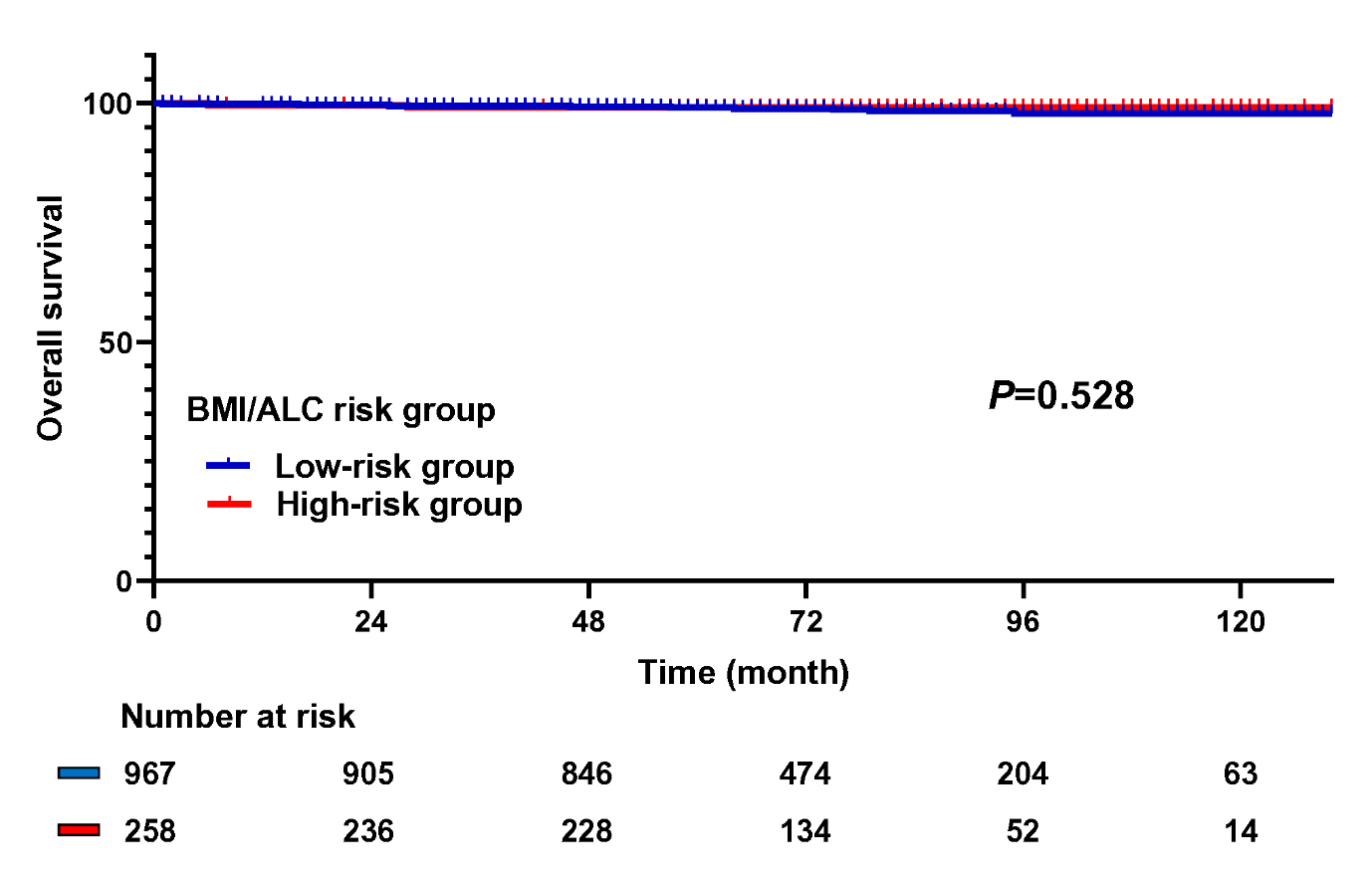


**Supplementary Figure S5.** Kapan–Meier survival curve for OS according to the BMI/ALC risk stratification groups (log-rank *P*=0.528)

ALC, absolute lymphocyte count; BMI, body mass index; OS, overall survival

**Supplementary Table S1.** CBC results of breast cancer patients and healthy females

|  | All patients | | | Body mass index, kg/m^2^ (%) | | | | | | | | |
| --- | --- | --- | --- | --- | --- | --- | --- | --- | --- | --- | --- | --- |
|  |  |  |  | <18.5 | | | 18.5-23.0 | | | ≥23.0 | | |
|  | Patients | Healthy female | *P*-value | Patients | Healthy female | *P*-value | Patients | Healthy female | *P*-value | Patients | Healthy female | *P*-value |
| Total (%) | 1,225  (100) | 35,991  (100) |  | 49  (4.0) | 2,964  (8.2) |  | 546  (44.6) | 19,271  (53.5) |  | 630  (51.4) | 13,756  (38.2) |  |
| WBC^*^  (range) | 6.16  (0.98-17.70) | 5.66  (1.69-19.75) | <0.001 | 5.92  (2.81-16.06) | 5.37  (1.76-19.75) | 0.014 | 5.90  (2.36-17.70) | 5.46  (1.69-19.26) | <0.001 | 6.41  (0.98-15.90) | 6.00  (2.26-17.13) | <0.001 |
| ALC^*^  (range) | 1.81  (0.34-4.92) | 1.89  (0.33-7.18) | <0.001 | 1.51  (0.78-2.52) | 1.79  (0.51-4.30) | <0.001 | 1.70  (0.57-3.73) | 1.82  (0.33-7.18) | <0.001 | 1.92  (0.34-4.92) | 2.02  (0.54-5.67) | <0.001 |
| ANC^*^ (range) | 3.82  (0.24-15.64) | 3.21  (0.24-15.64) | <0.001 | 3.92  (1.32-13.72) | 3.05  (0.64-17.61) | <0.001 | 3.68  (1.20-15.64) | 3.11  (0.33-17.61) | <0.001 | 3.93  (0.24-14.06) | 3.39  (0.76-14.40) | <0.001 |
| NLR  (range) | 2.34  (0.44-36.47) | 1.80  (0.24-23.98) | <0.001 | 2.97  (0.91-11.82) | 1.83  (0.30-15.51) | <0.001 | 2.37  (0.51-15.02) | 1.82  (0.24-20.72) | <0.001 | 2.27  (0.44-36.47) | 1.78  (0.30-23.98) | <0.001 |
| Monocyte^*^ (range) | 0.32  (0.01-1.18) | 0.31  (0.02-1.61) | 0.413 | 0.30  (0.12-0.62) | 0.29  (0.07-1.37) | 0.947 | 0.30  (0.05-0.72) | 0.30  (0.02-1.22) | 0.702 | 0.32  (0.01-1.18) | 0.33  (0.06-1.61) | 0.360 |
| Platelet^*^ (range) | 266.6  (69.0-548.0) | 266.6  (17.0-955.0) | 0.990 | 261.2  (158.0-498.0) | 253.4  (61.0-803.0) | 0.370 | 258.2  (69.0-548.0) | 261.5  (32.0-849.0) | 0.186 | 274.4  (82.0-527.0) | 276.5  (17.0-955.0) | 0.392 |

*****×10^3^/uL

ALC, absolute lymphocyte count; ANC, absolute neutrophil count; CBC, complete blood count; NLR, neutrophil-to-lymphocyte ratio; WBC, white blood cell

**Supplementary Table S2.** Surgery and adjuvant treatment for breast cancer patients

|  | Body mass index, kg/m^2^ (%) | | | | *P*-value |
| --- | --- | --- | --- | --- | --- |
|  | All patients | <18.5 | 18.5–23.0 | ≥23.0 |  |
| Breast surgery |  |  |  |  | 0.587 |
| Total mastectomy | 556 (45.4) | 25 (51.0) | 252 (46.2) | 279 (44.3) |  |
| Breast conserving surgery | 669 (54.6) | 24 (49.0) | 294 (53.8) | 351 (55.7) |  |
| Axillary surgery |  |  |  |  | 0.465 |
| SLNB | 1007 (82.2) | 43 (87.8) | 452 (82.8) | 512 (81.3) |  |
| ALND | 218 (17.8) | 6 (12.2) | 94 (17.2) | 118 (18.7) |  |
| Endocrine therapy |  |  |  |  | 0.293 |
| Not performed | 379 (30.9) | 14 (28.6) | 172 (31.5) | 193 (30.6) |  |
| Performed | 841 (68.7) | 35 (71.4) | 374 (68.5) | 432 (68.6) |  |
| Unknown | 5 (0.4) | 0 (0.0) | 0 (0.0) | 5 (0.8) |  |
| Chemotherapy |  |  |  |  | 0.583 |
| Not performed | 485 (39.6) | 24 (49.0) | 221 (40.5) | 240 (38.1) |  |
| Performed | 737 (60.2) | 25 (51.0) | 324 (59.3) | 388 (61.6) |  |
| Unknown | 3 (0.2) | 0 (0.0) | 1 (0.2) | 2 (0.3) |  |
| Radiotherapy |  |  |  |  | 0.841 |
| Not performed | 492 (40.2) | 22 (44.9) | 214 (39.2) | 256 (40.6) |  |
| Performed | 727 (59.3) | 27 (55.1) | 330 (60.4) | 370 (58.7) |  |
| Unknown | 6 (0.5) | 0 (0.0) | 2 (0.4) | 4 (0.6) |  |
| Anti-HER2 therapy |  |  |  |  | 0.257 |
| Not performed | 983 (80.2) | 43 (87.8) | 442 (81.0) | 498 (79.0) |  |
| Performed | 235 (19.2) | 6 (12.2) | 103 (18.9) | 126 (20.0) |  |
| Unknown | 7 (0.6) | 0 (0.0) | 1 (0.2) | 6 (1.0) |  |

ALND, axillary lymph node dissection; HER2, human epidermal growth factor receptor 2; SLNB, sentinel lymph node biopsy

**Supplementary Table S3.** Distribution of disease events in breast cancer patients

|  | All patients | Body mass index, kg/m^2^ (%) | | |  | Absolute lymphocyte count, ×10^3^/uL (%) | |
| --- | --- | --- | --- | --- | --- | --- | --- |
|  |  | <18.5 | 18.5–23.0 | ≥23.0 |  | Low | High |
| Total event | 152 | 5 (3.6) | 48 (34.8) | 85 (61.6) |  | 88 (63.8) | 50 (36.2) |
| Recurrence | 87 | 5 (5.7) | 31 (35.6) | 51 (58.6) |  | 56 (64.4) | 31 (35.6) |
| Locoregional | 32 | 2 (6.3) | 12 (37.5) | 18 (56.3) |  | 24 (75.0) | 8 (25.0) |
| Distant | 55 | 3 (5.5) | 19 (34.5) | 33 (60.0) |  | 32 (58.2) | 23 (41.8) |
| Secondary malignancy | 51 | 0 (0.0) | 17 (33.3) | 34 (66.6) |  | 32 (62.7) | 19 (37.3) |
| Contralateral breast cancer | 6 | 0 (0.0) | 1 (16.7) | 5 (83.3) |  | 2 (33.3) | 4 (66.6) |
| Thyroid cancer | 19 | 0 (0.0) | 9 (47.4) | 10 (52.6) |  | 14 (73.7) | 5 (26.3) |
| Lung cancer | 14 | 0 (0.0) | 3 (21.4) | 11 (78.6) |  | 9 (64.3) | 5 (35.7) |
| Stomach cancer | 4 | 0 (0.0) | 2 (50.0) | 2 (50.0) |  | 2 (50.0) | 2 (50.0) |
| Colorectal cancer | 1 | 0 (0.0) | 1 (100.0) | 0 (0.0) |  | 1 (100.0) | 0 (0.0) |
| Other cancer | 7 | 0 (0.0) | 1 (14.3) | 6 (85.7) |  | 4 (57.1) | 3 (42.9) |
| Death | 14 | 1 (7.1) | 8 (57.1) | 5 (35.7) |  | 10 (71.4) | 4 (28.6) |

**Supplementary Table S4.** Univariate and multivariable Cox regression analysis of OS

|  | Univariate analysis | | Multivariable analysis | |
| --- | --- | --- | --- | --- |
|  | HR (95% CI) | *P-*value | HR (95% CI) | *P*-value |
| Age^*^ | 1.00 (0.94–1.04) | .686 |  |  |
| Body mass index |  |  |  |  |
| 18.5–23.0 | Ref.^**^ |  |  |  |
| <18.5 | 1.40 (0.17–11.10) | 0.757 |  |  |
| ≥23.0 | 0.55 (0.18–1.68) | 0.294 |  |  |
| WBC count^*^ | 1.07 (0.82–1.38) | 0.629 |  |  |
| ALC^*^ | 0.41 (0.14–1.25) | 0.116 |  |  |
| ANC^*^ | 1.14 (0.90–1.45) | 0.264 |  |  |
| NLR^*^ | 1.08 (0.95–1.22) | 0.249 |  |  |
| Histologic grade |  |  |  |  |
| Low | Ref. |  |  |  |
| Intermediate | 2.69 (0.32–22.37) | 0.360 |  |  |
| High | 5.63 (0.69–45.79) | 0.106 |  |  |
| Nuclear grade |  |  |  |  |
| Low | Ref. |  |  |  |
| Intermediate | NE | NE |  |  |
| High | NE | NE |  |  |
| Estrogen receptor |  |  |  |  |
| Negative | Ref. |  |  |  |
| Positive | 0.54 (0.19–1.55) | 0.252 |  |  |
| Progesterone receptor |  |  |  |  |
| Negative | Ref. |  |  |  |
| Positive | 0.58 (0.20–1.64) | 0.301 |  |  |
| HER2 |  |  |  |  |
| Negative | Ref. |  |  |  |
| Positive | 2.49 (0.84–7.40) | 0.102 |  |  |
| Ki-67 LI, % |  |  |  |  |
| <14 | Ref. |  | Ref. |  |
| ≥14 | 8.06 (1.80–36.04) | 0.006 | 4.64 (1.01–21.27) | 0.048 |
| Tumor size, mm |  |  |  |  |
| ≤20 | Ref. |  | Ref. |  |
| >20 | 11.63 (2.60–51.98) | 0.001 | 7.77 (1.66–36.50) | 0.009 |
| Positive lymph node, count |  |  |  |  |
| 0 | Ref. |  | Ref. |  |
| 1–3 | 0.70 (0.15–3.13) | 0.657 | 0.50 (0.10–2.32) | 0.366 |
| ≥4 | 5.19 (1.56–17.25) | 0.007 | 2.09 (0.61–7.12) | 0.239 |

^*^Continuous variable

^**^Reference value

ALC, absolute lymphocyte count; ANC, absolute neutrophil count; CI, confidence interval; HER2, human epidermal growth factor receptor 2; HR, hazard ratio; LI, labelling index; NE, not estimated; NLR, neutrophil-to-lymphocyte ratio; Ref, reference; OS, overall survival; WBC, white blood cell
